# cGAS-STING pathway expression correlates with genomic instability and immune cell infiltration in breast cancer

**DOI:** 10.1038/s41523-023-00609-z

**Published:** 2024-01-02

**Authors:** Mengting Chen, Shibo Yu, Tineke van der Sluis, Mieke C. Zwager, Carolien P. Schröder, Bert van der Vegt, Marcel A. T. M. van Vugt

**Affiliations:** 1 Department of Medical Oncology, University of Groningen, University Medical Center Groningen, Groningen, The Netherlands; 2 Department of Pathology and Medical Biology, University of Groningen, University Medical Center Groningen, Groningen, The Netherlands; 3 Department of Medical Oncology, Netherlands Cancer Institute, Amsterdam, The Netherlands

**Keywords:** Breast cancer, Tumour heterogeneity

## Abstract

Genomic instability, as caused by oncogene-induced replication stress, can lead to the activation of inflammatory signaling, involving the cGAS-STING and JAK-STAT pathways. Inflammatory signaling has been associated with pro-tumorigenic features, but also with favorable response to treatment, including to immune checkpoint inhibition. In this study, we aim to explore relations between inflammatory signaling, markers of replication stress, and immune cell infiltration in breast cancer. Expression levels of cGAS-STING signaling components (STING, phospho-TBK1, and phospho-STAT1), replication stress markers (γH2AX and pRPA), replication stress-related proto-oncogenes (Cyclin E1 and c-Myc) and immune cell markers (CD20, CD4, and CD57) are determined immunohistochemically on primary breast cancer samples (n = 380). RNA-sequencing data from TCGA (n = 1082) and METABRIC (n = 1904) are used to calculate cGAS-STING scores. pTBK1, pSTAT1 expression and cGAS-STING pathway scores are all increased in triple-negative breast cancers compared to other subtypes. Expression of γH2AX, pRPA, Cyclin E1, c-Myc, and immune cell infiltration positively correlate with p-STAT1 expression (*P* < 0.001). Additionally, we observe significant positive associations between expression of pTBK1 and γH2AX, pRPA, c-Myc, and number of CD4+ cells and CD20+ cells. Also, cGAS-STING scores are correlated with genomic instability metrics, such as homologous recombination deficiency (*P* < 0.001) and tumor mutational burden (*P* < 0.01). Moreover, data from the I-SPY2 clinical trial (n = 71) confirms that higher cGAS-STING scores are observed in breast cancer patients who responded to immunotherapy combined with chemotherapy. In conclusion, the cGAS-STING pathway is highly expressed in TNBCs and is correlated with genomic instability and immune cell infiltration.

## Introduction

Breast cancer is one of the most frequent types of cancer, and the second-most common cause of cancer-related death among women^1^. Triple-negative breast cancer (TNBC) is characterized by the absence of estrogen receptor (ER), progesterone receptor (PR), and human epidermal growth factor receptor-2 (HER2) expression. Due to lack of targeted therapy, TNBC is regarded as a “hard-to-treat” breast cancer subtype and is associated with poor prognosis^2^.

Importantly, TNBCs are characterized by high levels of genomic instability, which can be caused by various factors, including defective homologous recombination (HR) repair or oncogene-induced replication stress^3,4^. Oncogene activation can lead to replication stress in various ways, including deregulation of the RB/E2F pathway, increased origin firing, depletion of the dNTP pool, and increased formation of DNA-RNA hybrids, known as R-loops^5^. For instance, overexpression of the proto-oncogene *CCNE1*, encoding Cyclin E1, which among breast cancers primarily occurs in TNBCs, has been shown to induce replication stress, mitotic aberrancies, and genomic instability^6^. In line with this notion, Cyclin E1 expression showed significant associations with expression of replication stress markers phospho-RPA32 (Ser33) and γH2AX in TNBC^7^.

Recently, it was demonstrated that genomic or chromosomal instability in cancer cells results in activation of the innate immune response, mediated by the cGAS-STING-TBK1 pathway, which subsequently leads to JAK/STAT signaling^8^. Mechanistically, when (fragments of) chromosomes end up in the cytoplasm after mitosis, the cytosolic DNA sensor cGAS (cyclic GMP-AMP synthase) is activated, which leads to the synthesis of cGAMP (2’,3’-cyclic GMP-AMP)^9^. cGAMP subsequently binds the adapter protein STING (stimulator of interferon genes), resulting in the phosphorylation of TBK1. In turn, TBK1 recruits and phosphorylates the transcription factor IRF3 (interferon regulatory factor 3) or NF-κB, thereby activating a type I interferon (IFN) response. The subsequent release of pro-inflammatory cytokines activates downstream JAK/STAT signaling^9^. Indeed, genomic instability, for instance caused by BRCA1 or BRCA2 deficiency, leads to mitotic missegregation of chromosome fragments and triggers cGAS-STING signaling^10,11^. Moreover, activation of cGAS-STING-STAT1 signaling upon BRCA2 inactivation was further potentiated upon PARP inhibition^12^. Likewise, treatment with chemotherapeutic agents that target DNA replication^13^ have also been shown to induce chromosome fragments in the cytoplasm and induce cGAS-STING signaling^14^.

Multiple studies suggested anti-tumor effects of STING-mediated immune pathways in several types of cancer. For instance, perinuclear-localized STING in ER^+^ breast cancers has been demonstrated to be an independent predictor of favorable prognosis, associated with higher immune cell infiltration and upregulation of immune checkpoints^15^. In line with this observation, DNA damage response-deficient breast tumors showed higher CD4^+^ and CD8^+^ lymphocytic infiltration^13^. Similarly, STING pathway activation in non-small cell lung cancer (NSCLC) predicted response to immunotherapy and was enhanced by cisplatin treatment^16^. In line with this notion, STING agonists showed potential anti-tumor effects in several types of cancer^17,18^.

Importantly, the JAK/STAT1 signaling that is induced upon cGAS-STING activation has been associated with response to treatment in patients with breast cancer, including response to immunotherapy or chemotherapy. For instance, chemotherapy-induced activation of the IFN/STAT1 pathway was associated with treatment response in ER^-^ breast cancer^19^. Phosphorylation of STAT1 at Ser727 was positively correlated with expression of programmed death-ligand 1 (PD-L1) and HLA class I, and could potentially serve as a biomarker to predict response to immunotherapy^20^. Moreover, in ER^+^ breast cancers, activation of the IFN signaling pathway has been associated with intrinsic resistance to CDK4/6 inhibitors and immune checkpoint activation^21^. Mechanistically, genome-wide genetic screens showed that interferon (IFN) signaling by tumor cells is a determinant of response to PD-L1 inhibitor^22,23^.

Clearly, cancer-intrinsic interferon signaling is associated with genomic instability and is relevant to treatment response. However, opposing roles of interferon signaling have been described^24^, with transient activation of inflammatory signaling inducing anti-tumor effects, whereas chronic activation may lead to tumor progression^25,26^. Therefore, a better understanding is required of the tumor types that show inflammatory pathway activation, to ultimately improve patient selection for immunotherapy or targeted therapy.

In this study, we investigated the clinical significance of inflammatory signaling and its associations with markers of replication stress, metrics of genome instability, and immune cell infiltration level in breast cancer samples. We analyzed the expression of key components of the cGAS-STING signaling pathway and their relation to replication stress markers, replication stress-inducing oncogenes, and immune cell markers in breast cancer patients. In parallel, we investigated the correlation between cGAS-STING inflammatory signaling and different molecular breast cancer subtypes, and its association with different genomic instability metrics using data from the TCGA and METABRIC cohorts. Finally, data from the I-SPY2 immunotherapy cohort was used to assess the involvement of cGAS-STING inflammatory signaling as a predictive factor for the response of breast cancer patients to immunotherapy.

## Results

### cGAS-STING signaling is higher in TNBCs

**Fig. 1:**
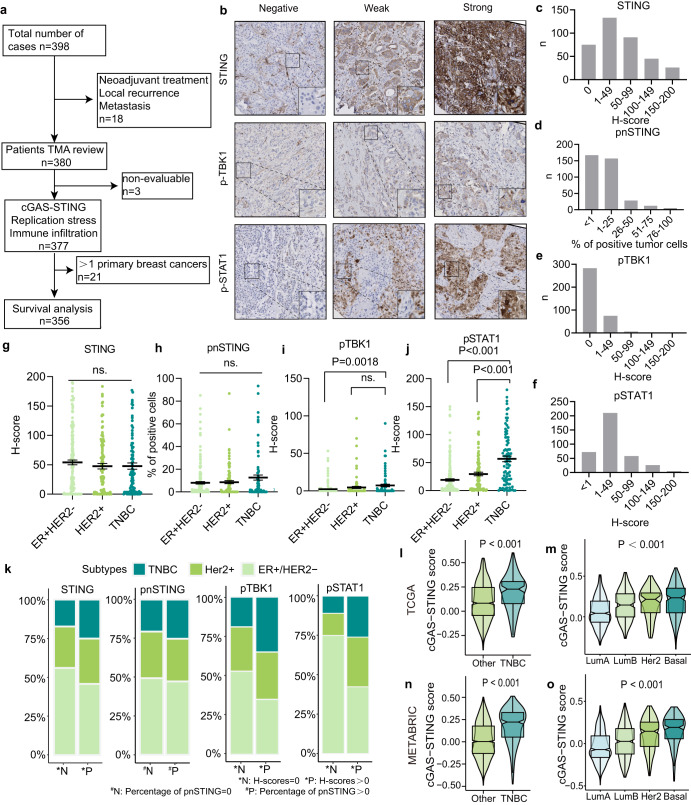
The cGAS-STING pathway is higher expressed in TNBC patients. **a** Flow diagram of sample selection. **b** Representative images of STING, pTBK1, and pSTAT1 staining in the breast cancer TMA. H-score distributions of STING (**c**), percentage of peri-nuclear STING (pnSTING) (**d**), H-score distributions of pTBK1 (**e**), and pSTAT1 (**f**) in tumor cells in breast cancer patient TMAs. Comparison of STING (**g**), pnSTING (**h**), pTBK1 (**i**), and pSTAT1 (**j**) expression among different breast cancer subtypes. Error bars represent mean ± SEM. Statistical significance was tested by Kruskal–Wallis test. **k** The stacked histograms are indicated of the different distributions of breast cancer subtypes between positive and negative samples for STING, pnSTING, pTBK1, and pSTAT1. Comparison of cGAS-STING scores among breast cancer patients with different molecular subtypes (**l**) and PAM50 subtypes (**m**) from the TCGA cohort. The bottom and top of the boxes reflect the 25th and 75th percentiles; Statistical significance was tested by Wilcoxon rank-sum test and Kruskal–Wallis test respectively. Comparison of cGAS-STING scores among breast cancer patients with different molecular subtypes (**n**) and PAM50 subtypes (**o**) in the METABRIC cohort. The bottom and top of the boxes reflect the 25th and 75th percentiles; Statistical significance was tested by Wilcoxon rank-sum test and Kruskal–Wallis test respectively.

We analyzed a cohort of 380 breast cancer samples (Fig. 1a). The clinicopathological characteristics and treatment of the patients in this cohort are summarized in Table 1. The protein expression levels of STING, pTBK1 (Ser172), and pSTAT1 (Ser727) were evaluated using immunohistochemistry (IHC) staining. Expression levels were quantified using H-scores (Fig. 1b–f). Percentage of perinuclear STING (pn)STING was quantified as a proxy of activated STING^15^ (Supplementary Fig. 1). As expected from components within a shared pathway, pSTAT1 expression was significantly associated with pnSTING (Spearman r = 0.157, *P* = 0.002) and pTBK1 (Spearman r = 0.339, *P* < 0.001) expression, across all breast cancer samples (Supplementary Table 1).

**Table 1:** Overview of the breast cancer patient cohort in this study.

		Total(%)	ER^+^/HER2^−^ (%)	HER2^+^ (%)	TNBC (%)
	Total	380	182	107	91
Menopausal status	Premenopausal	97 (25.7)	36 (19.9)	31 (29.5)	30 (33.0)
	Perimenopausal	38 (10.0)	17 (9.4)	12 (11.4)	9 (9.9)
	Postmenopausal	185 (48.7)	93 (51.4)	53 (50.5)	39 (42.9)
	Unknown	57 (15.0)	35 (19.3)	9 (8.6)	13 (14.3)
Histological grade	I	75 (19.7)	68 (37.4)	5 (2.7)	2 (2.2)
	II	125 (32.9)	76 (41.8)	34 (21.8)	15 (16.5)
	III	178 (46.8)	38 (20.9)	67 (62.6)	73 (80.2)
	Unknown	2 (0.6)	0 (0)	1 (0.9)	1 (1.1)
T	T1	231 (61.3)	124 (68.5)	58 (54.7)	49 (54.4)
	T2	131 (34.7)	54 (29.8)	42 (39.6)	35 (38.9)
	T3	10 (2.7)	3 (1.7)	5 (4.7)	2 (2.2)
	T4	5 (1.3)	0 (0)	1 (0.9)	4 (4.4)
N	N0	254 (68.3)	129 (72.5)	67 (63.2)	58 (65.9)
	N1	71 (19.1)	32 (18.0)	23 (21.7)	16 (18.2)
	N2	31 (8.3)	10 (5.6)	12 (11.3)	9 (10.2)
	N3	16 (4.3)	7 (3.9)	4 (3.8)	5 (5.7)
Stage	I	182 (48.9)	101 (57.1)	44 (41.5)	37 (41.6)
	II	140 (37.6)	59 (33.3)	46 (43.4)	35 (39.3)
	III	50 (13.4)	17 (9.6)	16 (15.1)	17 (19.1)
Chemotherapy	No	172 (45.3)	120 (65.9)	23 (21.5)	29 (31.9)
	Yes	208 (54.7)	62 (34.1)	84 (78.5)	62 (68.1)
Radiotherapy	No	107 (28.2)	55 (30.2)	22 (20.6)	30 (33.0)
	Yes	271 (71.3)	127 (69.8)	83 (77.6)	61 (67.0)
	Unknown	2 (0.5)	0 (0)	2 (1.8)	0 (0)

In terms of breast cancer subtypes, the total STING expression and percentage of pnSTING^+^ cells did not significantly differ among the three subtypes (*P* = 0.819 and *P* = 0.403 respectively, Fig. 1g, h). Notably, expression of pTBK1 was higher in TNBC compared to ER^+^/HER2^-^ cases (*P* = 0.0018), but similar to HER2^+^ cases (*P* = 0.174, Fig. 1i). pSTAT1 expression was also significantly higher in TNBC cases compared to ER^+^/HER2^-^ (*P* < 0.001) and HER2^+^ cases (*P* < 0.001, Fig. 1j). The percentage of cases that expressed pTBK1 and pSTAT was also higher in TNBC (Fig. 1k).

To compare our observations to large publicly available cohorts, we subsequently calculated a cGAS-STING activation score through analysis of a seven-gene mRNA expression signature, reflecting key components in the cGAS-STING pathway (*C6orf150*, *CCL5*, *CXCL10*, *IRF3*, *TBK1*, *TMEM173*, and *STAT1*)^27^ (further referred to as “cGAS-STING score”) (Supplementary Fig. 1a, b, Supplementary Table 2). We found that cGAS-STING scores were higher in the advanced-stage clinical subgroups in the METABRIC cohort, especially stage II and III&IV patients and patients with lymph node metastases (*P* < 0.001, Supplementary Fig. 3f, g). However, cGAS-STING scores were not related to tumor size (Supplementary Fig. 3e, *P* = 0.38). In the TCGA cohort of patients with breast cancer, cGAS-STING scores were not related to different clinical subgroups (tumor size, *P* = 0.075, Supplementary Fig. 3b; clinical stage, *P* = 0.23, Supplementary Fig. 3c; lymph node states, *P* = 0.684, Supplementary Fig. 3d). However, cGAS-STING scores were significantly higher in TNBCs, basal-like and HER2-enriched breast cancer subtypes, both in the TCGA (*P* < 0.001, Fig. 1l, m) and METABRIC cohorts (*P* < 0.001, Fig. 1n, o). Moreover, cGAS-STING scores were higher in cases with higher tumor grades (i.e., NPI2, NPI3, G2, and G3 subgroups) in the METABRIC cohort (*P* < 0.001, Supplementary Fig. 3h, i). Together, these observations indicate that TNBCs show elevated levels of cGAS-STING signaling.

### cGAS-STING signaling is associated with expression of replication stress-inducing oncogenes in breast cancer

**Fig. 2:**
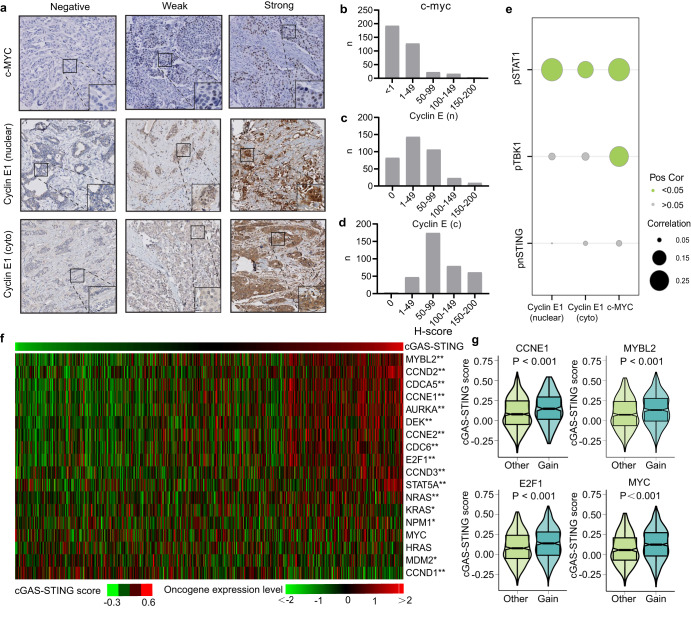
cGAS-STING pathway is correlated with replication stress-related oncogenes in breast cancer patients. **a** Representative images of c-Myc, Cyclin E1 (nuclear), and Cyclin E1 (cyto) IHC staining of breast cancer TMAs. H-score distributions of c-Myc (**b**), Cyclin E1 (nuclear) (**c**) and Cyclin E1 (cyto) (**d**) in breast cancer TMAs. **e** Spearman correlation analysis between cGAS-STING-related genes and replication stress-related oncogenes in our breast cancer cohort. The size of each circle represents the Spearman correlation co-efficiency, and the color of the circle represents positive or negative correlation with or without statistical significance. **f** Heatmap showing oncogene expression in breast cancer samples, ranked from right to left by the cGAS-STING score in the TCGA database. (* adjusted *P* < 0.05, ** adjusted *P* < 0.01). **g** Association between cGAS-STING scores and copy-number gain of replication stress-related oncogenes, from the TCGA database. The bottom and top of the boxes reflect the 25th and 75th percentiles; Statistical significance was tested by Wilcoxon rank-sum test.

Replication stress can facilitate of tumorigenesis and can be induced by oncogene activation^5^. Therefore, we next investigated whether inflammatory signaling was related to expression of proto-oncogenes, which were previously shown to induce replication stress when overexpressed. We focused on the Cyclin E1 and c-Myc proto-oncogenes, as they were established to induce replication stress in experimental models^28^. Moreover, overexpression of Cyclin E1 or Myc results in unscheduled origin firing within gene bodies and leads to replication-dependent DNA lesions^29,30^. In line with these data, our previous analysis of breast cancers demonstrated that Cyclin E1 expression was significantly correlated with expression of replication stress markers γH2AX and pRPA32^7^. We analyzed the expression of Cyclin E1 and c-Myc in relation to pSTAT1 expression in our breast cancer cohort, and observed that pSTAT1 expression was positively associated with the levels of both nuclear and cytoplasmic Cyclin E1 (nuclear Cyclin E1: Spearman r = 0.295, *P* < 0.001; cytoplasmic Cyclin E1: r = 0.176, *P* < 0.001). Also, a positive correlation was found between expression of pSTAT1 and c-Myc (r = 0.296, *P* < 0.001). pTBK1 levels were also positively associated with c-Myc (r = 0.241, *P* < 0.001), but not with Cyclin E1 expression (nuclear Cyclin E1: Spearman r = 0.062, *P* = 0.245; cytoplasmic Cyclin E1: r = 0.066, *P* = 0.216). Surprisingly, pnSTING expression was not significantly correlated with these two oncogenes in our cohort (Table 2; Fig. 2a–e).

**Table 2:** Spearman correlation between inflammatory signaling activation and replication stress markers and relative oncogenes among overall samples and different subtypes.

		γH2AX	pRPA	Cyclin E1 (n)	Cyclin E1 (c)	c-Myc
Overall						
pSTAT1	correlation	0.326	0.219	0.295	0.176	0.296
	*P* value	**<0.0001**	**<0.0001**	**<0.0001**	**0.001**	**<0.0001**
pTBK1	correlation	0.127	0.111	0.062	0.066	0.241
	*P* value	**0.018**	**0.035**	0.245	0.216	**<0.0001**
pnSTING	correlation	0.006	−0.010	0.044	0.049	0.056
	*P* value	0.914	0.854	0.403	0.359	0.294
ER^+^/HER2^−^						
pSTAT1	correlation	0.146	0.269	0.173	0.030	0.269
	*P* value	0.051	**<0.001**	**0.022**	0.693	**<0.001**
pTBK1	correlation	0.171	0.000	−0.097	−0.097	0.287
	*P* value	**0.022**	0.997	0.201	0.200	**<0.0001**
pnSTING	correlation	0.093	−0.012	−0.017	0.050	0.007
	*P* value	0.219	0.877	0.828	0.51609	0.926
HER2^+^						
pSTAT1	correlation	0.209	0.226	0.022	0.046	0.103
	*P* value	**0.032**	**0.021**	0.823	0.642	0.295
pTBK1	correlation	−0.012	0.276	0.041	−0.035	0.075
	*P* value	0.904	**0.005**	0.680	0.727	0.445
pnSTING	correlation	−0.111	0.004	0.112	0.059	−0.008
	*P* value	0.259	0.971	0.256	0.553	0.932
TNBC						
pSTAT1	correlation	0.176	0.213	0.280	−0.180	−0.029
	*P* value	0.137	**0.047**	**0.012**	0.108	0.806
pTBK1	correlation	−0.062	0.126	0.060	0.058	0.159
	*P* value	0.611	0.257	0.608	0.618	0.188
pnSTING	correlation	−0.151	0.011	−0.151	−0.100	−0.047
	*P* value	0.207	0.923	0.183	0.379	0.691

*P* values in bold indicate *P* < 0.05.

We next explored the correlation between cGAS-STING scores and expression of proto-oncogenes^31^ in the TCGA cohort. We found that increased mRNA expression of the majority of oncogenes was accompanied with higher cGAS-STING scores (Fig. 2f). Also, we found that genomic gain of various replication stress-related oncogenes was correlated to higher cGAS-STING scores in the TCGA cohort (Fig. 2g, *P* < 0.001). In summary, our results indicate that cGAS-STING inflammatory signaling is associated with the expression levels of replication stress-inducing oncogenes.

### cGAS-STING signaling is associated with genomic instability in breast cancer

**Fig. 3:**
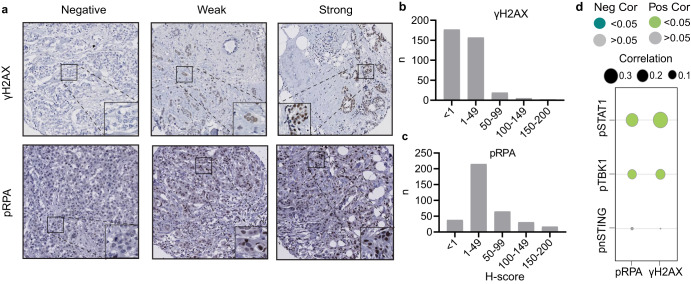
Higher cGAS-STING scores are associated with replication stress markers in breast cancer. **a** Representative images of γH2AX and pRPA staining in breast cancer TMAs. H-score distributions of γH2AX (**b**) and pRPA (**c**) in breast cancer TMAs. **d** Spearman correlation analysis of cGAS-STING-related genes versus replication stress markers in our own breast cancer patient cohort. The size of each circle represents the spearman correlation co-efficiency and the color of the circle represents positive or negative correlation with or without statistical significance.

To address whether cGAS-STING signaling was related to levels of genomic instability in breast cancer samples, we immunohistochemically analyzed replication stress markers γH2AX and phospho-RPA32 (Ser33) in our breast cancer cohort (Fig. 3a–c). Importantly, we found that in our overall cohort, pSTAT1 and pTBK1 expression was positively associated with both γH2AX (Spearman correlation pSTAT1: r = 0.326, *P* < 0.001; pTBK1: r = 0.127, *P* = 0.018) and pRPA expression (pSTAT1: r = 0.219, *P* < 0.001 pTBK1: r = 0.111, *P* = 0.035). Again, pnSTING expression was not significantly correlated with replication stress markers in our cohort (Table 2; Fig. 3d).

Next, we analyzed the associations in the individual breast cancer subgroups (Table 2). Positive correlations were observed between pSTAT1 and pRPA expression in all the breast cancer subgroups (r = 0.269, *P* < 0.001 in ER^+^/HER2^-^ cases, r = 0.226, *P* = 0.021 in HER2^+^ cases and r = 0.213, *P* = 0.047 in TNBC). pSTAT1 was also associated with nuclear Cyclin E1 in ER^+^/HER2^-^ (r = 0.173, *P* = 0.022) in TNBC patients (r = 0.280, *P* = 0.012). In addition, pTBK1 expression was significantly correlated with γH2AX in ER^+^HER2^-^ patients (r = 0.171, *P* = 0.022) and pRPA in HER2^+^ cases (r = 0.224, *P* = 0.022), indicating that that pSTAT1 and pTBK1 expression were strongly correlated with genomic instability. We additionally studied the relation between pSTAT1 expression and markers of genomic instability using linear regression analysis (Table 3). The covariates from univariate analysis with *P* < 0.05 were included for multivariate analysis. In multivariate analysis, pSTAT1 expression was associated with γH2AX (β = 0.221, *P* < 0.001), pRPA (β = 0.151, *P* = 0.006) and c-Myc (β = 0.139, *P* = 0.023), but not Cyclin E1, when corrected for tumor subtype, stage and grade.

**Table 3:** Relation between pSTAT1 versus markers of genomic instability and clinicopathological characteristics among the study cohort.

pSTAT1		Univariate			Multivariate		
		Beta	95% CI	*P* value	Beta	95% CI	*P* value
Tumor subtypes	ER^+^/HER2^-^	Ref.			Ref.		
	HER2^+^	0.125	2.085–19.001	**0.015**	0.046	−5.278–12.442	0.427
	TNBC	0.420	28.602–46.639	**<0.0001**	0.134	−2.400–26.212	0.103
Tumor grade	I	Ref.			Ref.		
	II	0.106	−2.024–19.093	0.113	0.084	−3.607–16.195	0.212
	III	0.339	15.832–35.847	**<0.0001**	0.165	0.769–22.728	**0.036**
Tumor Stage	I	Ref.					
	II	−0.036	−11.232–5.566	0.508			
	III	0.043	−7.333–17.302	0.427			
γH2AX		0.369	0.401–0.687	**<0.0001**	0.221	0.157–0.495	**<0.0001**
pRPA		0.167	0.053–0.218	**0.001**	0.151	0.035–0.201	**0.006**
Cyclin E1(n)		0.264	0.140–0.313	**<0.0001**	0.067	−0.064–0.180	0.351
Cyclin E1(c)		0.170	0.054–0.218	**0.001**	−0.089	−0.175–0.039	0.214
c-Myc		0.260	0.172–0.392	**<0.0001**	0.139	0.020–0.273	**0.023**

*P* values in bold indicate *P* < 0.05.

**Fig. 4:**
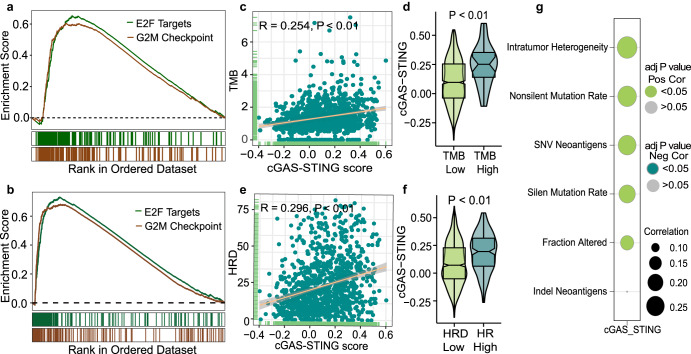
Higher cGAS-STING scores are associated with genomic instability in breast cancer. Enrichment plots for pathways that are related to genomic instability in the TCGA cohort (**a**) and METABRIC cohort (**b**). **c** Spearman correlation between cGAS-STING score and Tumor Mutation Burden score (log2 transformed) in the TCGA cohort. **d** The expression difference in cGAS-STING score between TMB high (TMB score å 10) and TMB low (TMB score < 10) subgroups in the TCGA cohort. The bottom and top of the boxes reflect the 25th and 75th percentiles; Statistical significance was tested by Wilcoxon rank-sum test. **e** Spearman correlation between cGAS-STING score and homologous recombination defects (HRD) score in the TCGA database. **f** Comparison of the cGAS-STING scores between HRD high (HRD score ≥ 42) and HRD low (HRD score < 42) subgroups in the TCGA database. The bottom and top of the boxes reflect the 25th and 75th percentiles; Statistical significance was tested by Wilcoxon rank-sum test. **g** Spearman correlation between cGAS-STING score, intratumor heterogeneity and different mutation-related scores in the TCGA database, including fraction altered, non-silent and silent mutation rate (log2 transformed), indel and single nucleotide variation (SNV) neoantigens (log2 transformed). The size of each circle represents the Spearman correlation co-efficiency, and the color of the circle represents positive or negative correlation with or without statistical significance.

The relation between cGAS-STING signaling and genomic instability was further explored in the TCGA and METABRIC cohorts. Gene Set Enrichment Analysis (GSEA) analysis was performed on data from both TCGA and METABRIC cohorts. Interestingly, both in the TCGA and METABRIC cohorts, cGAS-STING scores were associated with proliferation pathways, including ‘E2F targets’ and ‘G2/M checkpoint pathways’; genesets that were previously also associated with genomically unstable cancers^32,33^ (Fig. 4a, b, Supplementary Fig. 4a, b). We further analyzed the relationship between the cGAS-STING scores and different genomic instability markers in the TCGA cohort. We observed a positive correlation between cGAS-STING scores and tumor mutational burden (TMB, r = 0.254, *P* < 0.001), homologous recombination deficiency (HRD, r = 0.296, *P* < 0.001) and intratumor heterogeneity in the TCGA cohort (r = 0.28, *P* < 0.001) (Fig. 4c, e, Supplementary Fig. 4i). A similar correlation between TMB and cGAS-STING score was found in the METABRIC cohort (r = 0.0632, *P* < 0.01) (Supplementary Fig. 4o). According to previous studies^34–37^, clinically-used cut-offs to define high HRD (HRD ≥ 42) or high TMB (TMB ≥ 10) were used to further analyze the expression difference of the cGAS-STING score between high and low HRD or TMB groups. In line with our previous analysis, we observed that the cGAS-STING scores were higher in both TMB-High and HRD-High subgroups (TCGA: *P* < 0.001, Fig. 4d, f; METABRIC: *P* < 0.001, Supplementary Fig. 4p). In addition, a positive correlation between cGAS-STING score and several mutation-related metrics were observed in the TCGA cohort, including ‘fraction altered’ (r = 0.157, *P* < 0.001), ‘non-silent mutation rate’ (r = 0.256, *P* < 0.001), ‘silent mutation rate’ (r = 0.21, *P* < 0.001), ‘indels’ (r = 0.067, *P* = 0.0497), ‘single nucleotide variations’ (SNVs) and ‘neoantigens’ (r = 0.239, *P* < 0.001) (Fig. 4g, Supplementary Fig. 4j–n). Moreover, we observed a positive correlation between the cGAS-STING scores and somatic copy number alteration (sCNA) levels in the TCGA cohort (Supplementary Fig. 4c–h). Of note, the *BRCA1*-mutant samples (n = 18) showed significantly higher pSTAT1 expression compared to the wildtype cases, but not higher pnSTING and pTBK1 expression (n = 82, pnSTING, *P* = 0.141; pTBK1, *P* = 0.081; pSTAT, *P* = 0.003, Supplementary Fig. 5a–c). Since *BRCA2* mutation was found in only four samples with evaluable staining, these results were not included for analysis. In summary, our combined results show that cGAS-STING inflammatory signaling is elevated in breast cancers with genomic instability.

### cGAS-STING signaling is associated with immune cell infiltration

**Fig. 5:**
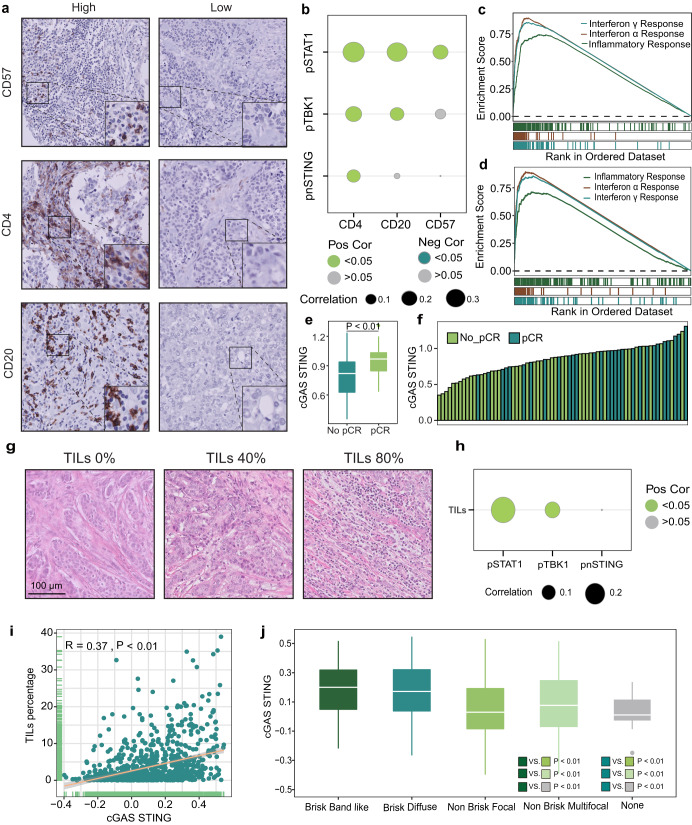
cGAS-STING signaling is associated with immune cell infiltration and response to PD-L1 inhibition combined with chemotherapy in breast cancer patients. **a** Representative images of CD57, CD4, and CD20 staining in breast cancer TMA. **b** Spearman correlation analysis between cGAS-STING-related genes and immune cell markers in our breast cancer TMA. The size of each circle represents the spearman correlation co-efficiency and the color of the circle represents positive or negative correlation with or without statistical significance. Enrichment plots for pathways that are related to immune process in the TCGA (**c**) and METABRIC (**d**) database. **e** Comparison of cGAS-STING scores between the pathologic complete response (pCR) group and the non-pCR group in the I-SPY2 cohort. The bottom and top of the boxes indicate the 25th and 75th percentiles; Statistical significance was tested by Wilcoxon rank-sum test. **f** The distribution of cGAS-STING scores in the durvalumab/olaparib arm from I-SPY2 cohort. **g** Representative images of H&E stainings with different percentages of tumor infiltrating lymphocytes (TILs). **h** Spearman correlation analysis between cGAS/STING-related genes and TILs in our patient cohort. The size of each circle represents the spearman correlation co-efficiency and the color of the circle represents positive or negative correlation with or without statistical significance. **i** Spearman correlation between cGAS-STING scores and percentage of TILs in the TCGA database. **j** Comparison of cGAS-STING scores among breast cancer patients with different TILs patterns in the TCGA database. The bottom and top of the boxes indicate the 25th and 75th percentiles; Statistical significance was tested by Kruskal–Wallis test.

STING pathway activation in tumor cells has been associated with response of patients to immunotherapy^16^. To address the relation between cGAS-STING signaling and immune cell infiltration, and also to address whether the observed cGAS-STING scores in bulk tumor sample analysis reflected tumor cells or infiltrated lymphocytes, we immunohistochemically analyzed the immune cell markers CD4 (T cell subset), CD20 (B cell marker), and CD57 (NK cell marker) in our breast cancer cohort (Fig. 5a). Notably, we found that in our overall cohort of breast cancers, a positive correlation was found between pSTAT1 expression and the number of all analyzed immune cell populations (CD4^+^: Spearman r = 0.369, *P* < 0.001; CD20^+^: r = 0.326, *P* < 0.001; CD57^+^: Spearman r = 0.188, *P* < 0.001). pTBK1 expression was positively associated with number of CD4^+^ (Spearman r = 0.212, *P* < 0.001) and CD20^+^ cells (Spearman r = 0.157, *P* < 0.05), but not with CD57^+^ cells (Spearman r = 0.095, *P* = 0.070). As for pnSTING, positive correlation was observed with CD4^+^ cell presence (Spearman r = 0.147, *P* < 0.01), but not CD57^+^ (Spearman r = − 0.02, *P* = 0.699) or CD20^+^ cells (Spearman r = 0.038, *P* = 0.464) (Fig. 5b). Additionally, the tumor-infiltrating lymphocytes (TILs) levels were positively associated with pSTAT1 (Spearman r = 0.293, *P* < 0.001) and pTBK1 (Spearman r = 0.117, *P* < 0.05) expression, but not with pnSTING in our breast cancer cohort (Spearman r = 0.016, *P* = 0.763) (Fig. 5g, h). In addition, we found that the cGAS-STING scores were positively associated with immune-related pathways in both TCGA and METABRIC cohorts (Fig. 5c, d).

An important open question is whether HER2-low (IHC score 1+ or 2+/in situ hybridization [ISH]-negative) breast cancers should be considered as a separate subtype, especially after the emergence of novel antibody-drug conjugates (ADCs)^38–40^. We analyzed HER2-low breast cancer in our cohort and found that the expression of cGAS-STING and immune cell infiltration did not differ between HER2-0 and HER2-low patients (Supplementary Fig. 6).

In addition, we explored the levels of TILs in the TCGA cohort. We again found a positive correlation between the percentage of TILs and cGAS-STING scores (r = 0.37, *P* < 0.01; Fig. 5i, j). Specifically, the cGAS-STING score showed a relative higher expression in the “brisk diffuse” and “brisk, band-like” subtypes, when compared to the non-brisk groups (“non-brisk focal”: “non-brisk, multi-focal”, and “None”) (*P* < 0.01; Fig. 5j).

### Inflammatory signaling and prognosis of breast cancer patients

Next, we analyzed the prognosis of breast cancer patients in our cohort. 356 patients were included for survival analysis (Table 4). The median follow-up time of our cohort was 140.6 months (range: 2.7–179.2 months). High or low protein expression of pSTAT1, pTBK1, and STING were divided by median score. High pSTAT1 expression was associated with pre-menopausal status (*P* = 0.008), higher histological grade (*P* < 0.001), larger tumor size (*P* = 0.038, Supplementary Table 3) and higher Ki-67 percentage (*P* < 0.001, Supplementary Fig. 3a). pTBK1 expression was also associated with higher tumor grade (*P* = 0.004, Supplementary Table 3) and Ki-67 percentage (*P* < 0.001, Supplementary Fig. 3a). Conversely, high STING expression was associated with lower N stage (*P* = 0.003).

**Table 4:** Univariate and multivariate COX regression analysis of pSTAT1 of breast cancer-specific survival (BCSS) based on clinical parameters and pSTAT1 expression.

		Univariate			Multivariate		
		HR	95% CI	*P* value	HR	95% CI	*P* value
Tumor subtypes	ER^+^/HER2^−^	Ref.			Ref.		
	HER2^+^	0.997	0.368–2.703	0.995	1.961	0.569-6.761	0.286
	TNBC	3.358	1.512–7.459	**0.003**	4.131	1.089–15.676	**0.037**
Tumor grade	I	Ref.			Ref.		
	II	1.876	0.378–9.313	0.442	2.090	0.391–11.171	0.389
	III	5.117	1.2–21.816	**0.027**	5.225	0.977–27.960	0.053
Stage	I	Ref.			Ref.		
	II	1.94	0.780–4.824	0.154	3.862	1.217–12.256	**0.022**
	III	6.268	2.560–15.347	**<0.001**	3.846	1.256–11.782	**0.018**
Chemotherapy	No	Ref.					
	Yes	1.219	0.525–2.829	0.645			
Radiotherapy	No	Ref.					
	Yes	0.677	0.319–1.440	0.311			
pSTAT1	Low	0.328	0.145–0.745	**0.008**	0.644	0.244–1.702	0.375
	High	Ref.			Ref.		

*P* values in bold indicate *P* < 0.05.

Univariate and multivariate Cox regression models were used to analyze the associations between pSTAT1 and patient survival (Table 4). In univariate analysis, lower pSTAT1 expression was associated with favorable breast cancer-specific survival (BCSS, HR: 0.328, 95% CI: 0.145–0.745, *P* = 0.008). Tumor subtypes, lower grade and lower stage were also associated with favorable BCSS, which were included in the multivariate analysis. However, pSTAT1 did not predict BCSS in the multivariate analysis (HR: 0.644, 95% CI: 0.244–1.702, *P* = 0.375). STING and pTBK1 expression were not associated with BCSS. STING, pTBK1 and pSTAT1 expression also did not predict relapse-free survival (RFS) in our cohort (Supplementary Table 4). These results indicate that STING, pTBK1, and pSTAT1 were not independent prognostic markers in our cohort.

To further explore whether cGAS-STING signaling was associated with the response of breast cancer patients to immune checkpoint inhibition, we analyzed data from I-SPY2 study^41^. We observed that in the durvalumab/olaparib arm, the cGAS-STING scores were higher in patients with pathologic complete response (pCR, *P* < 0.001, Fig. 5e, f). In summary, analysis of our own TMA and publicly available data showed that cGAS-STING inflammatory signaling was correlated with higher immune cell infiltration and better response to immune checkpoint inhibitor treatment in breast cancer patients.

## Discussion

In this study, we showed that the cGAS-STING pathway was differentially activated in different breast cancer subtypes, with TNBCs showing the highest activation. Moreover, pSTAT1 expression was positively associated with replication stress markers even after correction for clinical features. pTBK1 expression was also associated with γH2AX, pRPA and c-Myc expression. Furthermore, pSTAT1 and pTBK1 expression was associated with more aggressive tumor features, probably because the expression was higher in TNBC. Meanwhile, the cGAS-STING scores derived from publicly available cohorts showed significant positive correlations with the HRD, TMB, and SCNA. More importantly, we validated that the cGAS-STING pathway was associated with higher immune cell infiltration in breast cancer. Our observations show that inflammatory signaling is highly activated in genomically unstable breast cancers (Supplementary Fig. 7).

Similar cGAS-STING scores have been used to investigate the correlation with tumor immune microenvironment features. In oral squamous cell carcinoma (n = 327), combined high expression of cGAS and STING has been associated with immune cell infiltration and the expression profiles of immune-related genes^27^. Likewise, STING-related genes (CXCL10, CCL5, CGAS) were associated with immune activation in lung adenocarcinoma^16^. However, one of the drawbacks of using bulk transcriptomic data is that expression of cGAS-STING pathway components may reflect immune cell infiltration, rather than intrinsic cGAS-STING signaling in tumor cells. Therefore, we conducted IHC analysis of tumor tissue in our own patient’s cohort for further analysis.

*CCNE1* is amplified in approximately 9% of basal-like breast cancer^42^ and has been reported to induce replication stress^43^. Interestingly, cytoplasmic Cyclin E1 was reported to predict breast cancer recurrence and response to neoadjuvant chemotherapy^44,45^. Both nuclear and cytoplasmic Cyclin E1 expression were independently associated with γH2AX in breast cancer^7^. Therefore, we scored the nuclear and cytoplasmic Cyclin E1 separately, and found that tumors with higher nuclear Cyclin E1 showed higher pSTAT1 expression.

Higher STING expression was found to be associated with lower lymph node metastasis. Our result is in line with a study which showed that STING activity is a suppressor of metastatis^46^. Surprisingly, STING expression was not associated with replication stress markers in our cohort, which might be due to several reasons. Firstly, STING was reported to be phosphorylated upon activation^16,47^. Specifically, upon CHK1 inhibition or olaparib treatment, levels of phosphorylated STING (S366) were higher, while total STING expression did not increase^47^. Since we did not analyze phospho-STING levels, we may have missed these effects in our analysis. Secondly, STING is activated by cGAMP at the endoplasmic reticulum (ER), in which STING forms tetramers and translocates to ER-Golgi intermediate compartments. At the Golgi, the palymitolyation of STING has been shown to recruit TBK1 and IRF3^9^. Therefore, the cGAS-STING signature score probably better reflects the activation status of cGAS-STING signaling compared to STING levels alone.

Due to the limited response rate and efficacy of immunotherapy in breast cancer, it is of significant importance to identify patient subgroups that may respond to immune checkpoint inhibitors, and find useful biomarkers for selection^48^. pSTAT1 expression was reported as a potential biomarker for anti‑PD‑1/anti‑PD‑L1 immunotherapy for breast cancer^20^. In addition, one of the interferon-β-related cytokines, CXCL10, was shown to potentiate immune checkpoint blockade therapy in HR-deficient breast cancer^49^. Another reason to study the cGAS-STING and interferon signaling is that it may sensitize cancer patients to immunotherapy. For instance, targeting replication stress with CHK1 inhibitor promoted cGAS-STING signaling and NKT cell immune responses, and led to tumor regression^50^. Moreover, the STING agonist enhances the efficacy of PD-L1 monoclonal antibody in breast cancer immunotherapy by activating the interferon-β signaling pathway^51^. Our results also showed that *BRCA1*-mutant cancers exhibited higher cGAS-STING scores and higher pSTAT1 expression. The combination treatment with PARP inhibitor with PD-L1 inhibitor has been tested in *BRCA*-mutated metastatic breast cancer in phase 1/2 clinical trials^52^. Nevertheless, the combination treatment does not seem to be advantageous over PARP inhibitor alone^52,53^. Therefore, our results may facilitate efforts of identifying patients that may benefit from immunotherapy, PARP inhibition or combined treatment.

Of note, c-Myc was reported to suppress cGAS-STING mediated immune signaling^11^. Importantly, MYC was shown to promote immune suppression in TNBC through suppression of IFN signaling^11,54^. In addition, *MYC* amplification and overexpression led to low immune infiltration and cytolytic activity through suppression of interferon signaling and via activating of the transcription of DNMT1^55^. However, we found that pSTAT1 and pTBK1 were positively associated with c-Myc in ER^+^HER2^-^ tumors but not in TNBC. We argue that this might be due to several reasons. Firstly, Myc-induced DNA replication stress may lead to activation of STAT1 or TBK1. Secondly, the inflammatory signaling may in return regulate Myc activity. For instance, STAT1 has been shown to upregulate Myc and function as a pro-survival gene in serous papillary endometrial cancers^56^. TBK1 can promote Myc-dependent survival pathways in acute myeloid leukemia^57^. Future studies are needed to investigate the mechanisms in ER^+^HER2^-^ breast cancer.

In terms of patient prognosis, low pSTAT1 expression was associated with longer BCSS among the overall population which may be explained by the pro-tumorigenic effects of chronic inflammation^25^. However, pSTAT1 was not an independent prognostic marker in multivariate analysis. In fact, phosphorylation of STAT1 (Tyr701) has been associated with advanced tumor stage and worse survival in premenopausal breast cancer patients^58^. Whereas transient activation of inflammatory signaling can induce anti-tumor effects, chronic activation may lead to tumor progression^25,26^. Specifically, activation of the cGAS-STING pathway along with downstream non-canonical NF-κB signaling induced by chromosomal instability have been shown to drive metastasis^25^. Also, cGAS-dependent IL-6 secretion and IL6R signaling have recently been demonstrated to provide pro-survival signals in cancer cells, including TNBCs^59^. Therefore, the adverse effects of chronic inflammation should not be neglected.

In conclusion, our results showed an interplay between tumor intrinsic genomic instability and cGAS-STING innate immune signaling as well as the downstream STAT1 signaling. We also validated that higher cGAS-STING signaling is associated with higher immune cell infiltration. Further studies are still needed to elucidate the level of immune cell infiltration in CCNE1 overexpressed or other kinds of genomically unstable breast cancer. Our findings may potentially support identification of tumors which respond favorably to genotoxic chemotherapeutics or immunotherapy.

## Methods

### Breast cancer patients and tissue microarray

Consecutive primary breast tumor samples of HER2^+^, triple negative and the first 200 ER^+^HER2^−^ primary, non-metastasized, breast carcinomas diagnosed between 2006 and 2017 in the University Medical Center Groningen (UMCG, The Netherlands) were retrospectively collected and included in a tissue microarray (TMA). In line with Dutch law and UMCG security guidelines, the retrospective collection of clinicopathological characteristics and overall survival data from patient charts and the Personal Records Database was approved by the Local Ethics Review Board Pathology non-WMO studies (UMCG research register number 201900243, approved on 18-8-2020). This study was performed in line with the principles of the Declaration of Helsinki. Patients receiving neoadjuvant treatment, with local recurrence or metastasis at presentation were excluded. 18 samples were excluded after prior inclusion, resulting in a study population of 380 samples, including 182 ER^+^HER2^-^, 107 HER2^+^ and 91 TNBC cases. Patients with two primary breast cancers were excluded from the survival analysis. Tissue collection and storage of clinicopathological and follow up data was only performed upon approval of patients via informed consent. Clinical data was collected at the UMCG and stored digitally in a central database, which is solely accessible by two dedicated data managers. The specimens used in this study were obtained from redundant diagnostic material stored at the Department of Pathology, UMCG. No objection to research on redundant tissue was recorded from these patients in the institutional record of objection.

### Immunohistochemistry (IHC) staining

Slides (4 µm) of formalin-fixed and paraffin-embedded tissue were deparaffinized in xylene and rehydrated in decreasing ethanol solutions. Antigen retrieval was achieved by microwave heating for 15 min in 1 mM EDTA buffer pH 8 (10 mM Tris/EDTA buffer pH 9 for pTBK1 staining). Endogenous peroxidase was blocked by incubating in 0.3% H2O2 in phosphate buffered saline (PBS) solution for 30 min. After 1-h incubation with diluted primary antibody, a secondary goat-anti-rabbit-HRP (DAKO, Glostrup, Denmark, 1:100 in PBS/ 1% BSA/ 1% serum) was incubated for 30 min, followed by a rabbit-anti-goat-HRP (DAKO, 1:100 in PBS/1% BSA/1% serum) incubation for 30 min. The primary antibodies in this study include anti-STING (1:100, Cell Signaling #13647), anti-pSTAT1 (Ser727, 1:200, Cell Signaling, MA, USA, #8826S,) and anti-pTBK1 (1:50, Ser172, Cell Signaling #5483). Cyclin E (1:1000; rabbit, #sc-198, Santa Cruz Biotechnology), c-Myc (RTU, #790-4628, Roche), phospho-RPA32 (Ser33) (1:400, #A300-246A, Bethyl), γ-H2AX (1:300, #05-636, Millipore). Staining was visualized by using 3,3’-diaminobenzidine tetrahydochloride substrate (Sigma). For c-Myc and pRPA32 staining, the complete staining procedure was performed on an autostainer (BenchMark Ultra, Roche). The staining protocols for Cyclin E1, c-Myc, and γH2AX stainings were described previously^6^. For Ki-67 (30-9), cytokeratin-8/18 (B22.1&B23.1), CD4 (SP35), CD20 (L-26) and CD57 (NK-1) staining, the antibodies were pre-diluted by the manufacturer (Ventana) and sections were stained on a Benchmark Ultra immunostainer (Ventana) according to the manufacturer’s protocols.

### Image evaluation

Scoring was performed semi-quantitatively without knowledge of clinical data by two independent researchers (M.C. and S.Y.) and was supervised by a breast cancer pathologist (B.v.d.V.). IHC stainings were considered evaluable when a tumor core contained at least 10% tumor cells. Tumor cells were identified by morphology and cytokeratin-8/18 positivity. The H scores (range from 0 to 200) were calculated by intensity (negative: 0; medium: 1; high: 2) multiplied by the percentage of cells in each group. As previously described, peri-nuclear STING (pnSTING) expression was evaluated by the percentage of positive cells as a proxy for activated STING^15^. The percentage of pnSTING-positive tumor cells was then scored semi-quantitatively. Only the tumor cells with strong staining in perinuclear area (within 1 µm) were considered as pnSTING positive cells (Supplementary Fig. 1). In addition, the nuclear and cytoplasmic Cyclin E1 expression was scored separately according to the previous studies^7,44,60^. The proliferation marker Ki-67 and the CD4 and CD20 immune cell stainings were quantified by using Visiopharm Integrator System (VIS) (Visiopharm, Denmark) software, by using algorithms that have been previously validated^61,62^. For CD57, we manually counted positive cells. Tumor-infiltrating lymphocytes (TILs) were evaluated with hematoxylin and eosin (H&E) staining, according to an international guideline^63^. The staining scores from the individual cores of each tumor were averaged for analysis.

### TCGA, METABRIC, and I-SPY2 data

Gene expression data and clinical information of breast cancer patients in the TCGA and METABRIC cohorts were obtained from cBioportal and UCSC Xena on August 10th, 2022^42,64,65^. For the TCGA cohort, transcriptomic data, copy number data, clinical information, ER, PR, and HER2 status were obtained of 1082 breast cancers. For the METABRIC cohort, TMB, clinical data including stage, tumor size, lymph node metastases status, Nottingham Prognostic Index (NPI), histological grade, and PAM50 subtypes were obtained for 1904 breast cancers^66^. Also, ER, PR and HER2 status were also obtained for the METABRIC cohort. To investigate the association between cGAS-STING scores and genomic instability level in breast cancer patients, metrics of genomic instability, including homologous recombination deficiency (HRD) in the TCGA cohort were accessed from a previous study^67^. The percentage and patterns of TILs based on H&E images in TCGA was also approached through previous study to further explore the relation between cGAS-STING score and TILs^68^. The H&E staining images from TCGA were classified into 5 subtypes, namely “brisk, band-like group” (most TILs localized to the bordering of the tumor), “brisk diffuse group” (TILs scattered throughout >30% of the area of the tumor), “non-brisk focal group” (TILs scattered <5% but >1% of the area of the tumor), “non-brisk, multi-focal group” (TILs scattered <30% but >5% of the area of the tumor) and “none” (TILs involving <1% of the area of the tumor).

In order to explore the association between cGAS-STING scores and immunotherapy response in breast cancer patients, transcriptomic data from I-SPY2 trial were analyzed (n = 71)^41^. Original transcriptomic data and patient response data were accessed through GSE173839 in Gene Expression Omnibus (GEO; https://www.ncbi.nlm.nih.gov/geo/).

### cGAS-STING score and gene set enrichment analysis

We assembled genes involved in the cGAS-STING pathway from previous studies^27^ into a cGAS-STING score, including *C6orf150* (encoding cGAS/MB21D1), *CCL5*, *CXCL10*, *IRF3*, *TBK1*, *TMEM173* (encoding STING1) and *STAT1* (Supplementary Table 1). Single-sample Gene Set Enrichment Analysis (ssGSEA) was used to calculate the enrichment scores of these genes in the TCGA and I-SPY2 cohort. Of note, *TMEM173* (STING1) mRNA expression levels could not be retrieved for samples of the METABRIC database, therefore only *MB21D1*, *CCL5*, *CXCL10*, *IRF3*, *TBK1*, and *STAT1* were used to calculate the enrichment scores in the METABRIC cohort. Additionally, to explore difference between cGAS-STING^high^ and cGAS-STING^low^ groups, Gene Set Enrichment Analysis (GSEA) was performed using the Hallmarks gene sets^69^. We used the GSEA in these two cohorts by using the R package “clusterProfiler” according to the high and low cGAS-STING score^70^. The hallmarks gene sets (h.all.v7.5.1.symbols.gmt) from Molecular Signatures Database (MSigDB) were used to perform the enrichment analysis (https://www.gsea-msigdb.org/gsea/index.jsp). The pathways with a p.adjust value less than 0.05 were considered to be statistically significant.

### Copy number alteration (CNA) analysis and SCNA score

The gene-level CNA data for breast cancers in the TCGA database were downloaded from the cBioportal portal (https://www.cbioportal.org/). The CNA data were thresholded with the following cut-off: −2 = homozygous deletion; −1 = hemizygous deletion; 0 = neutral / no change; 1 = gain; 2 = high-level amplification. We combined gain and high-level amplification as CNA gain states for each oncogene in this study. Somatic copy number alterations (SCNA) levels, including “SCNA Level” “Chrom SCNA Level”, “Arm SCNA Level”, “Chrom arm SCNA Level” and “SCNA Level normalized by size” in TCGA for BRCA were accessed from a previous study^71^.

### Mutation-related score, HRD, and TMB

Mutation-related scores, including non-silent and silent mutation rate, indel and single nucleotide variation (SNV) neoantigens, fraction altered, intratumor heterogeneity, and homologous recombination defects (HRD) in TCGA for BRCA were collected from Thorsson et al. in the TCGA cohort^67^. HRD ≥ 42 was defined as high HRD according to a previous study^34^. Tumor mutation burden (TMB) data in the TCGA and METABRIC were collected from the cbioportal portal (TMB (nonsynonymous)) (https://www.cbioportal.org/) and according to previously published papers, TMB ≥ 10 was defined as high TMB in the study^35,36^.

### Statistical analysis

Data were analyzed using Statistical Package for Social Sciences version 23.0 (SPSS Inc.), R-4.0.4 and GraphPad Prism 8.4.2. Comparison of continuous variables among different groups was analyzed by Kruskal–Wallis tests or Wilcoxon rank-sum tests as indicated. Spearman correlation was used to compare the correlation of protein expression levels. Univariate and multivariate linear regression model was used to investigate the relation between pSTAT1 and markers of genomic instability. Univariate and multivariate regression analysis was performed using Cox proportional hazards model. The Benjamini–Hochberg method was used to adjust the *p*-value for multiple comparisons. Results were considered statistically significant when *P* < 0.05.

### Reporting summary

Further information on research design is available in the Nature Research Reporting Summary linked to this article.

### Supplementary information

SUPPLEMENTAL MATERIAL

reporting summary

## Data Availability

Gene expression data and clinical information from TCGA and METABRIC are downloaded from the cBioportal portal (https://www.cbioportal.org/) and UCSC Xena on August 10th, 2022. The GEO datasets can be downloaded through the accession number GSE173839 (https://www.ncbi.nlm.nih.gov/geo/). The data analyzed in the current study are available from the corresponding author upon reasonable request.

## Code availability

The codes used in this study are publicly available and listed in Supplementary Table 5.
