# Supplementary material for: cGAS-STING pathway expression correlates with genomic instability and immune cell infiltration in breast cancer
Source: NPJ Breast Cancer. 2024 Jan 2;10:1. doi: 10.1038/s41523-023-00609-z (PMC10761738; doi:10.1038/s41523-023-00609-z)
Supplement: Supplementary file 1 — SUPPLEMENTAL MATERIAL [file 41523_2023_609_MOESM1_ESM.pdf]

Supplementary information for:

Expression of the cGAS-STING pathway is associated with high levels of genomic instability and immune cell infiltration in breast cancer

Mengting Chen, Shibo Yu, Tineke van der Sluis, Mieke C. Zwager, Bert van der Vegt, Marcel A. T. M. van Vugt*

*corresponding author: m.vugt@umcg.nl

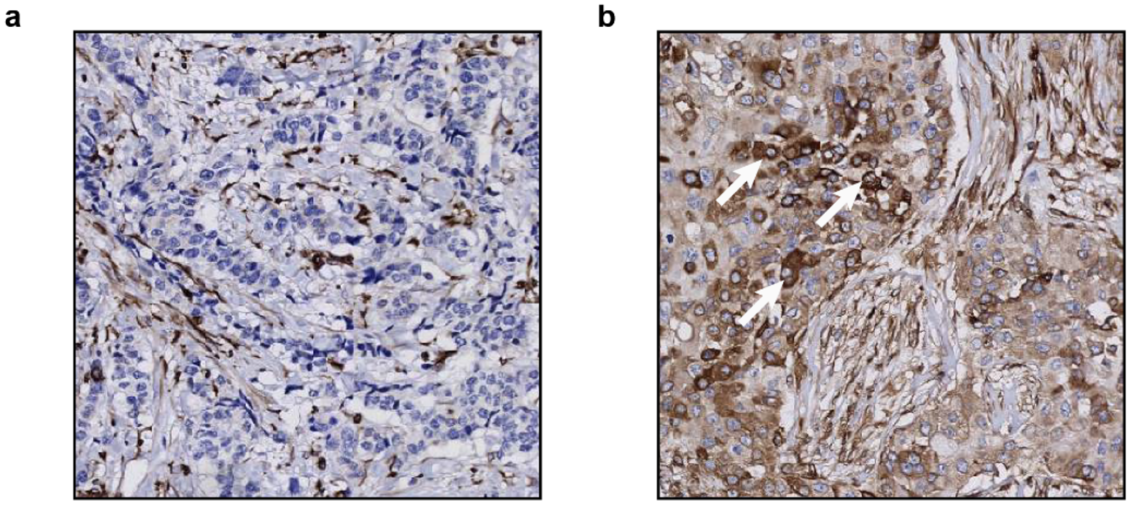

Supplementary Figure 1. Representative images of quantification of pnSTING tumor cells. (a) A TMA core with negative pnSTING expression. **(b)** A TMA core with positive pnSTING expression. White arrows represent pnSTING-positive cells.

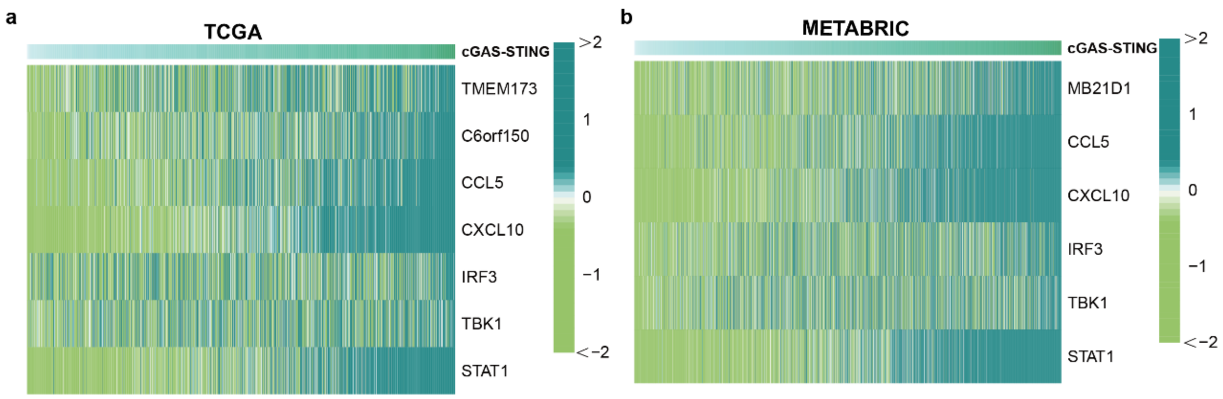

Supplementary Figure 2. The expression of genes which compose cGAS-STING score in breast cancer. (a, b) Heatmap of expression of cGAS-STING-related genes ranked by the cGAS-STING score in the TCGA (a) and METABRIC (b) database.

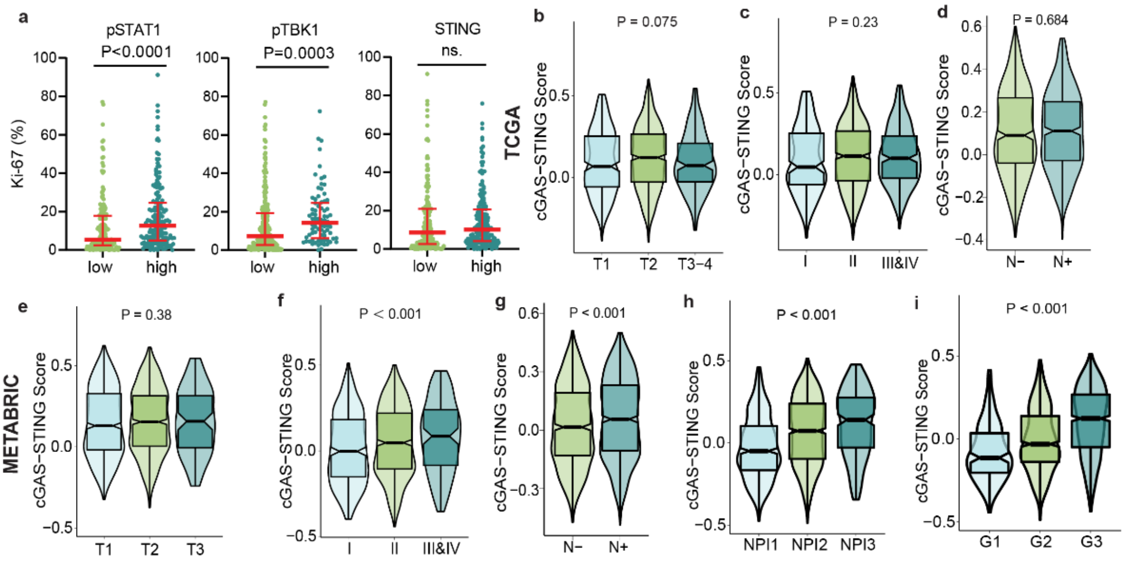

Supplementary Figure 3. Comparison of cGAS-STING signaling and patient clinical characteristics. (a) Comparison of percentage of Ki-67-positive cells in tumors with low or high cGAS-STING components**. (b-d)** Comparison of cGAS-STING scores among patients with different tumor size (b), tumor stage (c) and lymph node status (d) in the TCGA database. The bottom and top of the boxes indicate the 25th and 75th percentiles; Statistical significance was tested by Wilcoxon rank-sum test or Kruskal–Wallis test. **(e-i)** Comparison of cGAS-STING scores among patients with different tumor size (e), tumor stage (f), lymph node status (g), Nottingham prognostic index (h), histologic Grade (i) in the METABRIC cohort. The bottom and top of the boxes indicate the 25th and 75th percentiles; Statistical significance was tested by Wilcoxon rank-sum test or Kruskal–Wallis test.

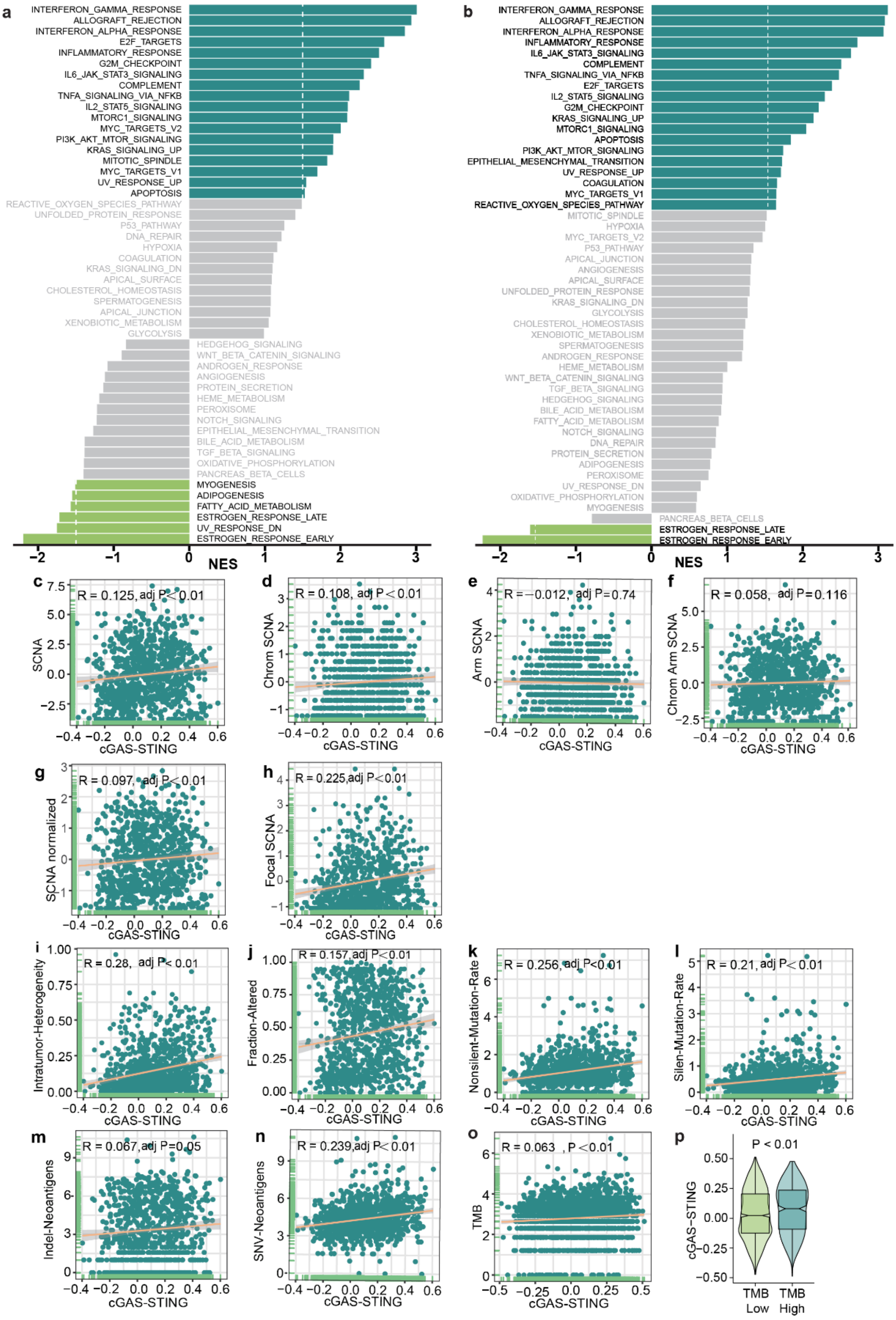

Supplementary Figure 4. Higher cGAS-STING score is associated with genomic instability in breast cancer. Figure legend continues on next page.

Supplementary Figure 4 (continued)

(a, b) GSEA enrichment results with Normalized Enrichment Scores (NES) of all biological pathways in the METABRIC (a) and TCGA (b) database, the dotted line indicates the threshold of NES >1.5 or <−1.5. **(c-h)** Spearman correlation between cGAS-STING score and different types of somatic copy number alterations (sCNAs) levels in the TCGA database. **(i-n)** Spearman correlation between cGAS-STING score, intratumor heterogeneity and different mutation-related scores in the TCGA database related to Fig. 4g. **(o)** Spearman correlation between cGAS-STING score and Tumor Mutation Burden (TMB) score (log2 transformed) in the METABRIC database. **(p)** The difference of cGAS-STING score between TMB high and TMB low subgroups in the METABRIC database. The bottom and top of the boxes are the 25th and 75th percentiles; Statistical significance was tested by Wilcoxon rank-sum test.

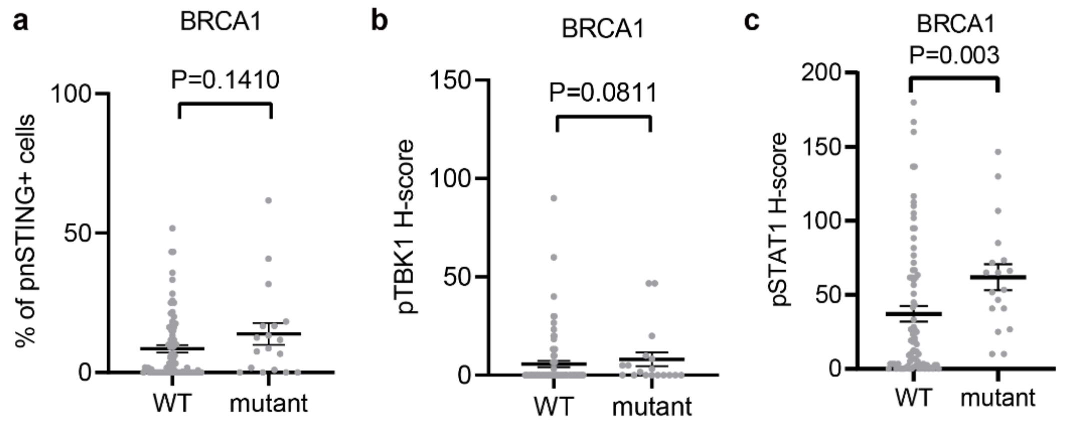

Supplementary Figure 5. The expression of inflammatory signaling based on BRCA1 status. The percentage of pnSTING$^+$ cells (**A**) and H-scores of pTBK1 (**B**) and pSTAT1 (**C**) in BRCA1 wildetype (WT, n=82) and mutant (n=18) breast cancer patients. Error bars represent mean ± SEM. Statistical significance was tested by Wilcoxon rank-sum test.

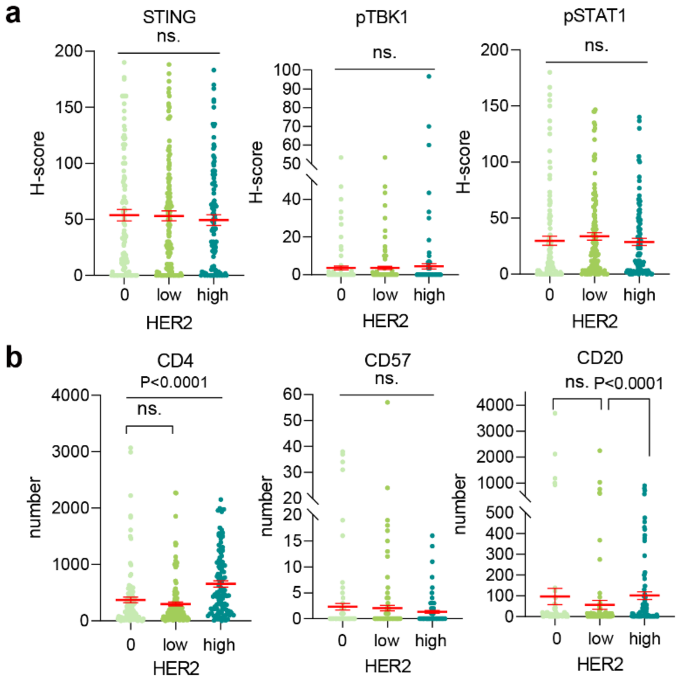

Supplementary Figure 6. Expression of cGAS-STING signaling components and immune cell infiltration does not differ between HER2-0 and HER2-low patients. **(a)** Comparison of cGAS-STING signaling expression among HER2-0, HER2-low and HER2-high (positive) breast cancers. **(b)** Comparison of immune cell infiltration among HER2-0, HER2-low and HER2-high (positive) breast cancer. Error bars represent mean ± SEM. Statistical significance was tested by Kruskal–Wallis test.

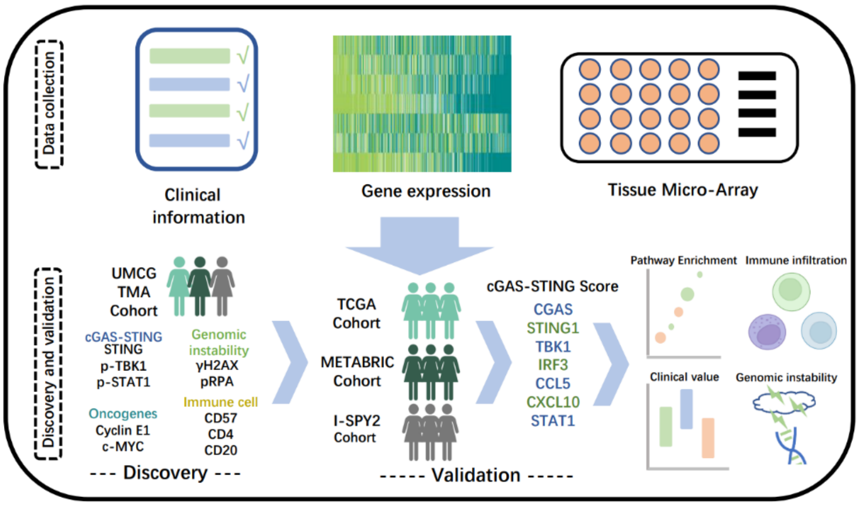

Supplementary Figure 7. Flowchart of this study. Evaluating the cGAS-STING Score in four independent cohorts (UMCG /TCGA/METABRIC/I-SPY2) and analyzing the characteristics of the cGAS-STING score in terms of pathway enrichment, immune infiltration, clinical value and genomic instability in breast cancer patients.

Supplementary Table 1. Spearman correlation among the inflammatory signaling proteins.

		pnSTING	pTBK1	pSTAT1
STING	correlation	0.810	0.024	0.054
	P value	**<0.001**	0.644	0.297
pnSTING	correlation	-	0.045	0.157
	P value		0.396	**0.002**
pTBK1	correlation	-	-	0.339
	P value			**<0.001**

Supplementary Table 2. Names and Entrez ID of cGAS-STING-related genes.

Gene symbol	Full name	Others gene symbol	Entrez ID
CGAS	Cyclic GMP-AMP Synthase	C6orf150/ MB21D1	115004
CCL5	C-C Motif Chemokine Ligand 5	TCP228	6352
CXCL10	C-X-C Motif Chemokine Ligand 10	IP-10/ GIP-10	3627
IRF3	Interferon Regulatory Factor 3	IIAE7/ IRF-3	3661
TBK1	TANK Binding Kinase 1	NAK	29110
STING1	Stimulator Of Interferon Response CGAMP Interactor 1	STING/ TMEM17	340061
STAT1	Signal Transducer And Activator Of Transcription 1	STAT91/ ISGF-3	6772

Supplementary Table 3. Relation between inflammatory signaling protein expression and patient clinicopathological characteristics.

		pSTAT1			pTBK1			STING		
		**low**	**high**	**P**	**low**	**high**	**P**	**low**	**high**	**P**
Menopausal status	Premenopausal	34	60	**0.008**	73	25	0.704	52	42	0.337
	Perimenopausal	19	18		29	9		16	20	
	Postmenopausal	92	91		139	37		84	99	
	Unknown	35	19		43	11		30	24	
Histological grade	I	48	24	**<0.001**	64	9	**0.004**	39	32	0.557
	II	68	57		103	22		60	65	
	III	65	107		115	52		83	89	
	Unknown	1	0		1	0		1	0	
T	T1	103	126	**0.038**	174	54	0.861	110	118	0.804
	T2	73	57		100	27		62	62	
	T3	5	2		6	21		4	4	
	T4	0	3		2	1		2	2	
N	N0	124	125	0.309	187	62	0.635	114	135	**0.003**
	N1	36	34		56	12		35	35	
	N2	13	17		23	6		24	6	
	N3	4	11		10	3		10	5	

Supplementary Table 4. Univariate COX regression analysis patient survival based on inflammatory signaling proteins.

	HR	95% CI	P value
**BCSS**			
STING			
low	1.007	0.478-2.121	0.986
high	Ref.		
pnSTING			
low	1.051	0.491-2.251	0.898
high	Ref.		
pTBK1			
low	0.424	0.174-1.036	0.06
high	Ref.		
**RFS**			
pSTAT1			
low	0.720	0.436-1.188	0.198
high	Ref.		
STING			
low	1.075	0.650-1.777	0.777
high	Ref.		
pnSTING			
low	1.029	0.588-1.605	0.910
high	Ref.		
pTBK1			
low	1.063	0.623-1.701	0.910
high	Ref.		

BCSS: breast cancer-specific survival; **RFS**: relapse-free survival

Supplementary Table 5. Published codes and publicly available tools used in this study.

Resource	Source	Related figures or data
R package, “pheatmap”	https://cran.r-project.org/web/packages/pheatmap/index.html	Heapmap
R package, “ggplot2”	https://cran.r-project.org/web/packages/ggplot2/index.html	Visualization of box plot, Violin plot, Bubble plot, Bar plot and Scatter plot
R package, “clusterProfiler”	https://bioconductor.org/packages/release/bioc/html/clusterProfiler.html	Enrichment analysis
R package, “Limma”	https://bioconductor.org/packages/release/bioc/html/limma.html	Differential expression analysis
R package, “GSVA”	https://www.bioconductor.org/packages/release/bioc/html/GSVA.html	ssGSEA was performed using GSVA to calculate the cGAS-STING enrichment score
