# Supplementary material for: cGAS-STING pathway expression correlates with genomic instability and immune cell infiltration in breast cancer
Source: NPJ Breast Cancer. 2024 Jan 2;10:1. doi: 10.1038/s41523-023-00609-z (PMC10761738; doi:10.1038/s41523-023-00609-z)
Supplement: Supplementary file 2 — reporting summary [file 41523_2023_609_MOESM2_ESM.pdf]

nature portfolio

Corresponding author(s): Marcel van Vugt, Bert van der Vegt

Last updated by author(s): Sep 18, 2023

Reporting Summary

Nature Portfolio wishes to improve the reproducibility of the work that we publish. This form provides structure for consistency and transparency in reporting. For further information on Nature Portfolio policies, see our Editorial Policies and the Editorial Policy Checklist.

Statistics

For all statistical analyses, confirm that the following items are present in the figure legend, table legend, main text, or Methods section.

n/a	Confirmed	
☐	☒	The exact sample size (*n*) for each experimental group/condition, given as a discrete number and unit of measurement
☐	☒	A statement on whether measurements were taken from distinct samples or whether the same sample was measured repeatedly
☐	☒	The statistical test(s) used AND whether they are one- or two-sided<br>*Only common tests should be described solely by name; describe more complex techniques in the Methods section.*
☐	☒	A description of all covariates tested
☐	☒	A description of any assumptions or corrections, such as tests of normality and adjustment for multiple comparisons
☐	☒	A full description of the statistical parameters including central tendency (e.g. means) or other basic estimates (e.g. regression coefficient) AND variation (e.g. standard deviation) or associated estimates of uncertainty (e.g. confidence intervals)
☐	☒	For null hypothesis testing, the test statistic (e.g. *F*, *t*, *r*) with confidence intervals, effect sizes, degrees of freedom and *P* value noted<br>*Give P values as exact values whenever suitable.*
☒	☐	For Bayesian analysis, information on the choice of priors and Markov chain Monte Carlo settings
☒	☐	For hierarchical and complex designs, identification of the appropriate level for tests and full reporting of outcomes
☐	☒	Estimates of effect sizes (e.g. Cohen's *d*, Pearson's *r*), indicating how they were calculated

Our web collection on statistics for biologists contains articles on many of the points above.

Software and code

Policy information about availability of computer code

Data collection	Public data were downloaded from https://www.cbioportal.org/.
Data analysis	Data were analyzed with SPSS 23.0, GraphPad Prism 8 or R-4.3.1.

For manuscripts utilizing custom algorithms or software that are central to the research but not yet described in published literature, software must be made available to editors and reviewers. We strongly encourage code deposition in a community repository (e.g. GitHub). See the Nature Portfolio guidelines for submitting code & software for further information.

Data

Policy information about availability of data

All manuscripts must include a data availability statement. This statement should provide the following information, where applicable:

- Accession codes, unique identifiers, or web links for publicly available datasets
- A description of any restrictions on data availability
- For clinical datasets or third party data, please ensure that the statement adheres to our policy

Gene expression data and clinical information from TCGA and METABRIC can be downloaded from the cBioportal (https://www.cbioportal.org/). Upon reasonable request, other data are available from the corresponding authors.

Research involving human participants, their data, or biological material

Policy information about studies with human participants or human data. See also policy information about sex, gender (identity/presentation), and sexual orientation and race, ethnicity and racism.

Reporting on sex and gender	This is a breast cancer study, and all the samples involved were from women.
Reporting on race, ethnicity, or other socially relevant groupings	This study does not specifically include or exclude participants from any race or ethnicity.
Population characteristics	Population characteristics are described in the manuscript.
Recruitment	This is a retrospective study and does not involve patient recruitment.
Ethics oversight	Approval was granted by the Local Ethics Review Board Pathology non-WMO studies (UMCG research register number 201900243, approved on 18-8-2020).

Note that full information on the approval of the study protocol must also be provided in the manuscript.

Field-specific reporting

Please select the one below that is the best fit for your research. If you are not sure, read the appropriate sections before making your selection.

☒ Life sciences ☐ Behavioural & social sciences ☐ Ecological, evolutionary & environmental sciences

For a reference copy of the document with all sections, see nature.com/documents/nr-reporting-summary-flat.pdf

Life sciences study design

All studies must disclose on these points even when the disclosure is negative.

Sample size	This study involves 380 breast cancer samples.
Data exclusions	Patients receiving neoadjuvant treatment, with local recurrence or metastasis at presentation were excluded. Patients with two primary breast cancers were excluded from the survival analysis.
Replication	Not applicable.
Randomization	Not applicable.
Blinding	Not applicable.

Reporting for specific materials, systems and methods

We require information from authors about some types of materials, experimental systems and methods used in many studies. Here, indicate whether each material, system or method listed is relevant to your study. If you are not sure if a list item applies to your research, read the appropriate section before selecting a response.

Materials & experimental systems

n/a	Involved in the study	
☐	☒	Antibodies
☒	☐	Eukaryotic cell lines
☒	☐	Palaeontology and archaeology
☒	☐	Animals and other organisms
☒	☐	Clinical data
☒	☐	Dual use research of concern
☒	☐	Plants

Methods

n/a	Involved in the study	
☒	☐	ChIP-seq
☒	☐	Flow cytometry
☒	☐	MRI-based neuroimaging

Antibodies

Antibodies used	STING (Cell Signaling 13647), pSTAT1 (Ser727, Cell Signaling, 8826S), pTBK1 (Ser172, Cell Signaling 5483), pRPA(Bethyl, A300-246A), Cyclin E (Santa Cruz Biotechnology, sc-198), c-Myc (Roche,790–4628),γ-H2AX (Millipore, 05–636)

The following antibodies were from Ventana: Ki-67 (30-9), cytokeratin-8/18 (B22.1&B23.1), CD4 (SP35), CD20 (L-26) and CD57 (NK-1)

Validation	γ-H2AX, pRPA, Cyclin E, c-Myc (PMID: 32964114), STING(PMID: 30297358) and pSTAT1(PMID: 31081058) antibodies have previously been tested and validated.
